# A Case of Crossed Left Renal Ectopia Identified during Colostomy Reversal

**DOI:** 10.1155/2017/3674603

**Published:** 2017-01-18

**Authors:** Rachel NeMoyer, Sumana Narayanan, Nell Maloney-Patel

**Affiliations:** ^1^Rutgers Robert Wood Johnson University Hospital, New Brunswick, NJ 08903, USA; ^2^Roswell Park Cancer Institute, Buffalo, NY 14263, USA; ^3^The Cancer Institute of New Jersey, New Brunswick, NJ 08903, USA

## Abstract

Unilateral crossed renal ectopia without fusion is an uncommon anatomic anomaly, which often goes undiagnosed. We report a case of this renal variant discovered incidentally during colostomy reversal after Hartmann's procedure for diverticular stricture.

## 1. Introduction

The genitourinary system has significant anatomic variability. However, unilateral crossed renal ectopia without fusion of the kidneys is an uncommon problem, occurring in one in 75,000 patients [1]. Many of these anomalies go unnoticed and may only be found at time of autopsy, although infection, stones, and trauma are known associated risk factors of an ectopic kidney. The ureteral course is frequently unpredictable when the kidney is in a nontraditional location [1]. Here, we present a case of a patient undergoing colostomy reversal who was found to have crossed renal ectopia with the ureter coursing in the presacral space.

## 2. Case Report

**Figure 1:**
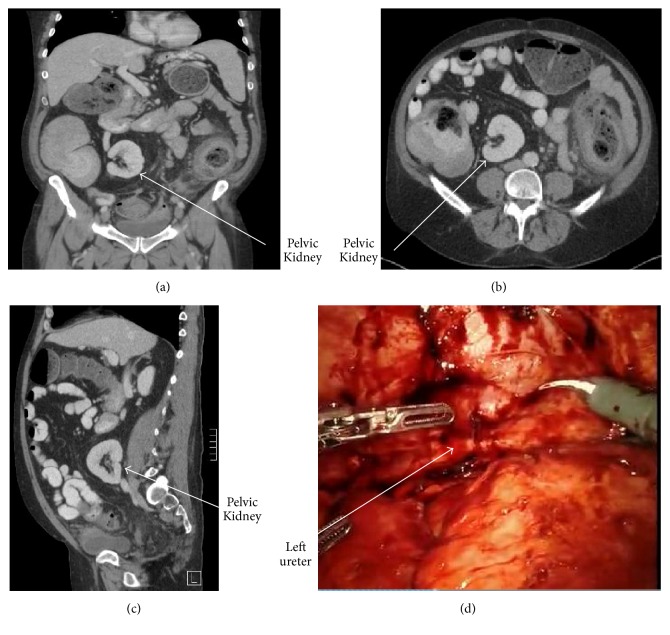
(a) Coronal view of unilateral crossed left renal ectopia without fusion to the right kidney. (b) Axial view of unilateral crossed left renal ectopia without fusion to the right kidney. (c) Sagittal view of unilateral crossed left renal ectopia without fusion to the right kidney. (d) Robotic view of dense adhesions to the left ureter in presacral space.

The patient is a 48-year-old male with a history of Hartmann's procedure for diverticular stricture causing large bowel obstruction. Permission was obtained from the patient to allow discussion and publication of his case. At the time of initial exploration, he was noted to have both kidneys on the right hand side with the left kidney crossing over and lying at approximately the level of the anterior superior iliac spine (ASIS) in the retroperitoneal space, as seen on pre-op CT (see Figures 1(a), 1(b), and 1(c)). Ureteral stents had been placed for the initial surgery due to the degree of inflammatory changes, but the ureters were not isolated at the time of Hartmann's procedure. The patient represented for colostomy reversal approximately 16 weeks later, again, with preoperative cystoscopy and ureteral stent placement. The operation was started robotically but was soon converted to an open laparotomy incision due to the complex pelvic anatomy and need for further adhesiolysis (see Figure 1(d)). In mobilizing the rectum to perform the colostomy reversal, the ureteral stent for the left ectopic kidney was palpated crossing behind the rectum in the presacral space. The ureter was completely identified along its course and care was taken to avoid injury. The colorectal anastomosis was then completed using the double purse-string technique. The Firefly device was used to ensure that there was good perfusion to the anastomosis and on-table colonoscopy was performed up to the level of the anastomosis with no air leak identified. The patient did well postoperatively and was seen at follow-up without complication.

## 3. Discussion

Crossed renal ectopia is a rare anatomic variant of the kidney. The majority of cases of crossed renal ectopia are asymptomatic and go undiagnosed until incidentally identified on imaging, during surgical exploration for unrelated reasons, or on autopsy. The most common form of crossed renal ectopia is the fused form accounting for approximately 90% of cases, with the unfused type (as was the case in our patient) accounting for the remaining 10% of cases [2].

Crossed renal ectopia has been commonly described as four different variants, including bilateral crossed renal ectopia, S-shaped kidney, L-shaped kidney, and disc kidney [3]. The most common variant is unilaterally fused kidneys with inferior ectopia. This occurs when the lower pole of the uncrossed kidney is fused to the upper pole of the crossed kidney. The second most common variant is the S-shaped, which involves the uncrossed kidney in its usual position and the crossed kidney inferior to the normal kidney without renal fusion, as is described in this case. The L-shape involves transverse positioning of the crossed kidney, with the lower poles in contact and the disc kidney occurs when medial borders of the uncrossed and crossed kidneys are fused together [2–4]. This becomes significant when determining the course of the ureter of the ectopic kidney during dissection of abdominal and pelvic structures in the operating room.

During colon and rectal surgery, injury to the ureter can occur due to the close proximity of the ureters to the colon. Although these injuries are relatively uncommon, when they occur they can cause significant patient morbidity. The typical course of the ureters begins at the ureteropelvic junction, posterior to the renal vein and artery. It then descends anteriorly on the psoas muscle. The left ureter is usually found near the descending and sigmoid colon while the right ureter is closely associated with the cecum, appendix, and ascending colon [5]. As the ureter enters the pelvis, it typically crosses anterior to the iliac vessels. This occurs at the bifurcation of the common iliac artery into the external and internal iliac arteries and can be used as a landmark for pelvic dissections. In this patient, due to the crossed renal ectopia, the course of the ureter was unclear.

There is scant information in the literature about methods to determine the course of the ureter preoperatively in cases with known genitourinary tract anomalies. Buyukdereli et al. [6] described using DMSA (Technetium-99m dimercaptosuccinic acid) dynamic renal scintigraphy as a possible imaging tool to preoperatively identify ureteral anatomy. Renal scintigraphy allows the physician to detect anatomic and/or functional abnormalities of the kidneys or urinary tract by assessing images obtained. Renal tubular cells predominantly incorporate DMSA; therefore, DMSA can detect and define pyelonephritis and renal cortical scar. It can also be used to assess the size, shape, position, and relative functional cortical mass of the kidneys, as well as define renal and collecting system anatomy [7]. By identifying the course of the ureters using a combination of imaging studies, the surgeon would have better awareness preoperatively as to the probable course of the ureter, in turn likely decreasing the chance of accidental injury. Ureteral stents have also been used to help identify the ureters during surgery and to quickly identify an intraoperative injury; however, stent placement has not been shown to prevent ureteral injury [8].

This patient was known to have crossed renal ectopia during his initial Hartmann's procedure as well as his colostomy reversal. Prior to the reversal, we may have considered obtaining further imaging to better determine the course of the aberrant ureter; however, there has been no consensus as to the best course of action in these patients as this is a rare entity. Further studies to identify any imaging modalities which may aid in preoperative visualization of the course of the ureters in patients with known renal anomalies may be useful in operative planning and may prevent ureteral injury.
